# Foot Assessment Clinical Scales in Charcot-Marie-Tooth Patients: A Scoping Review

**DOI:** 10.3389/fnhum.2022.914340

**Published:** 2022-06-24

**Authors:** Chiara Rambelli, Davide Mazzoli, Martina Galletti, Giacomo Basini, Paolo Zerbinati, Paolo Prati, Francesca Mascioli, Stefano Masiero, Andrea Merlo

**Affiliations:** ^1^Gait & Motion Analysis Laboratory, Sol et Salus Hospital, Rimini, Italy; ^2^Department of Neuroscience, Section of Rehabilitation, University of Padova, Padua, Italy; ^3^Neuro-Orthopedic Unit, Sol et Salus Hospital, Rimini, Italy

**Keywords:** Charcot-Marie-Tooth disease, foot assessment, clinical scales, metric properties, Foot Posture Index

## Abstract

**Introduction:**

Charcot-Marie-Tooth disease (CMT) is a slow and progressive peripheral motor sensory neuropathy frequently associated with the cavo-varus foot deformity. We conducted a scoping review on the clinical scales used to assess foot deviations in CMT patients and analyzed their metric properties.

**Evidence Acquisition:**

A first search was conducted to retrieve all scales used to assess foot characteristics in CMT patients from the Medline, Web of Science, Google Scholar, Cochrane, and PEDro databases. A second search was conducted to include all studies that evaluated the metric properties of such identified scales from the same databases. We followed the methodologic guidelines specific for scoping reviews and used the PICO framework to set the eligibility criteria. Two independent investigators screened all papers.

**Evidence Synthesis:**

The first search found 724 papers. Of these, 41 were included, using six different scales: “Foot Posture Index” (FPI), “Foot Function Index”, “Maryland Foot Score”, “American Orthopedic Foot & Ankle Society's Hindfoot Evaluation Scale”, “Foot Health Status Questionnaire”, Wicart-Seringe grade. The second search produced 259 papers. Of these, 49 regarding the metric properties of these scales were included. We presented and analyzed the properties of all identified scales in terms of developmental history, clinical characteristics (domains, items, scores), metric characteristics (uni-dimensionality, inter- and intra-rater reliability, concurrent validity, responsiveness), and operational characteristics (normative values, manual availability, learning time and assessors' characteristics).

**Conclusions:**

Our results suggested the adoption of the six-item version of the FPI scale (FPI-6) for foot assessment in the CMT population, with scoring provided by Rasch Analysis. This scale has demonstrated high applicability in different cohorts after a short training period for clinicians, along with good psychometric properties. FPI-6 can help health professionals to assess foot deformity in CMT patients over the years.

## Introduction

Charcot-Marie-Tooth (CMT) disease is a peripheral progressive motor sensory neuropathy. It represents the most frequent hereditary neuromuscular disorder with an estimated prevalence of up to 40 cases per 100,000 individuals (Martini et al., 1998; Pareyson and Marchesi, 2009). This pathological condition is characterized by the progressive deterioration of peripheral nervous system fibers, causing loss of both motor and sensory functions. Lower limb afflictions are often the earliest ones to arise, including distal muscle atrophy and weakness, which could result in foot drop, sensory loss, absent tendon reflexes, muscle cramps, and cavo-varus foot deformity. The average age of onset is between 10 and 20 years of age (Pareyson and Marchesi, 2009). The cavo-varus foot deformity usually represents the first clinical symptom of the disease. Consequently, the presence of bilateral cavus foot deformity in a healthy subject should be investigated for CMT when other etiologies have already been excluded (Stino et al., 2019). CMT patients exhibiting a foot deformity account for 71% of the total: in children aged 0–5 the planovalgus foot is the prevailing one, whereas in older patients cavo-varus associated with claw-toes and ankle instability is more widespread. Differences in timing and severity of muscle involvement cause an imbalance between agonistic and antagonistic muscles, resulting in a vicious circle of ensuing denervation and biomechanical alterations (Stino et al., 2019). Foot deformity, in association with muscle weakness, leads to the development of gait alterations typical of CMT, besides negatively affecting the patient's quality of life (Crosbie et al., 2008). Gait deviation also relates to limitations in everyday activities (Fulk et al., 2017; Mazzoli et al., 2019).

To properly assess foot characteristics in the CMT population, a scale should be designed for this kind of neurological condition. The design and validation of a clinical scale is a challenging and time-consuming process (Boateng et al., 2018). Identifying a set of items that reasonably describe the desired outcome is the first of a list of consecutive steps that must be followed and that deal with face and construct validity, internal consistency, item reduction and scaling, reliability, and validity (Boateng et al., 2018). These are commonly referred to as the metric properties of a scale.

In 2002 Razeghi and Batt critically reviewed the different methods used to classify foot types and observed a poor correlation between radiographic and observational indicators of foot morphology and its functional characteristics during walking (Razeghi and Batt, 2002). In the last decades, many different tools have been developed to assess foot characteristics in a wide spectrum of pathologies. Some of these were developed for purely surgical purposes, in order to aid with a radiographic evaluation of the ankle/foot complex injuries (Leigheb et al., 2016). Other tools were developed by clinicians or podiatrists to assess and monitor the patient's perceived disability in systemic chronic pathologies such as rheumatoid arthritis (Saag et al., 1996), milder afflictions of the foot like cutaneous and nail disorders (Bennett et al., 1998) and neurological conditions including CMT.

Although several clinical assessment tools were developed, to the best of our knowledge, no systematic or scoping review has been developed to identify the existing tools and their characteristics and metric properties. Therefore, the aim of this scoping review is collect all clinical scales used up till now for the clinical evaluation of the foot in CMT patients and to analyse their metric properties and characteristics to identify the most appropriate tool to be used with these patients.

## Materials and Methods

This scoping review was conducted and progressively updated until July 2021, in accordance with the JBI methodology guidelines for scoping reviews, which are an extension of PRISMA guidelines specific for scoping reviews (Tricco et al., 2018; Peters et al., 2020).

### Leading Questions

We followed a two-step procedure consistent with our two leading questions: (I) What were the clinical scales previously adopted in literature to assess foot characteristics in CMT patients? (II) What are their psychometric properties?

### First Step and Search Strategy

An initial limited search of Medline was undertaken to identify the appropriate articles. The text words contained in the titles and abstracts of relevant articles, and the index terms used to describe the articles were used to develop a first full search strategy for the Medline, Cochrane, PEDro, and Web of Science databases. Google Scholar was also considered. The search strategy, including all identified keywords and index terms, was adapted for each database. The reference list of all included findings was screened for additional studies to be included by manual search.

The first search strategy for the first step was: [(Charcot Marie Tooth) OR (Charcot Marie Tooth disease [MeSH Terms])] AND [(foot joints[MeSH Terms]) OR (ankle joints[MeSH Terms]) OR (ankle[MeSH Terms]) OR foot OR ankle] AND [assessment OR evaluation OR measure OR (outcome measure) OR scale OR (outcome assessment health care[MeSH Terms]) OR (disability evaluation[MeSH Terms])].

### Eligibility Criteria for the First Step

We used the PICO framework to set the eligibility criteria. Since two steps with two different searches were conducted, two different PICOs were set up.

#### Population

We included studies on CMT patients, both adults and children. No restrictions on study design were set. We included all types of studies such as reviews, experimental studies, and observational studies. Studies published both in English and Italian were included and no time limitations were set. Exclusion criteria were studies on animals, genetic studies, studies concerning other pathologies and withdrawn papers. Abstracts from conferences, where full text was not available or did not exist, were also excluded.

#### Intervention

Studies could involve any type of intervention.

#### Comparison

No limitations were set for this parameter.

#### Outcome

Papers had to use at least one clinical scale specific for the assessment of foot characteristics in the CMT population. Scales including foot assessment as part of an overall assessment of disease severity were not included.

### Second Step and Search Strategy

In the second stage, we analyzed the metric properties of the scales identified in Step 1. This was carried out through an advanced search on Medline, Cochrane, PEDro, Web of Science and Google Scholar by looking for the name of each scale found in the previous step and associated with the terms “validity”, “validation”, “consistency”, “reliability” and “responsiveness” in either the title or in the abstract, and excluding translated studies and cultural adaptations of the scale (“NOT translation”, “NOT adaptation”). Again, the bibliography of the included papers was screened to further identify papers to be included.

The second full search string was: (((scale name[Title/Abstract]) OR (scale acronym[Title/Abstract])) AND (foot[Title/Abstract])) AND (validity[Title/Abstract] OR validation[Title/Abstract] OR consistency[Title/Abstract] OR reliability[Title/Abstract] OR responsiveness[Title/Abstract]) NOT (fascia[Title/Abstract] OR fasciopathy[Title/Abstract] OR fasciitis) NOT (translation[Title/Abstract] OR adaptation[Title/Abstract] OR elderly[Title/Abstract] OR treatment[Title/Abstract] OR trial[Title/Abstract]).

### Eligibility Criteria for the Second Step

#### Population

We considered the populations on which metric properties of the included clinical scales had been analyzed. To focus on studies necessary to our aims – i.e. foot assessment CMT patients – we excluded those treating pathologies of the plantar fascia, hallux valgus and studies on the geriatric population.

#### Intervention

Studies had to involve the analysis of the metric properties of at least one clinical scale from those retrieved in the first step.

#### Comparison

No limitations were set for this parameter.

#### Outcome

Metric properties of the scales, such as inter/intra-rater reliability, concurrent validity, or internal consistency.

### Evidence Selection and Data Extraction

All identified citations were collected, and duplicates were removed. Titles and abstracts were screened, and potentially relevant sources were retrieved. Two independent reviewers (authors CR and AM) assessed the full text and decided whether to include selected papers. Reasons for exclusion of sources of evidence were recorded and reported in the scoping review. Any disagreements that arose between the reviewers at each given stage of the selection process was discussed. Relevant data from each paper were collected in tables and presented in a narrative synthesis.

## Results

### Scales Assessing Foot Characteristics in CMT Patients

**Figure 1:**
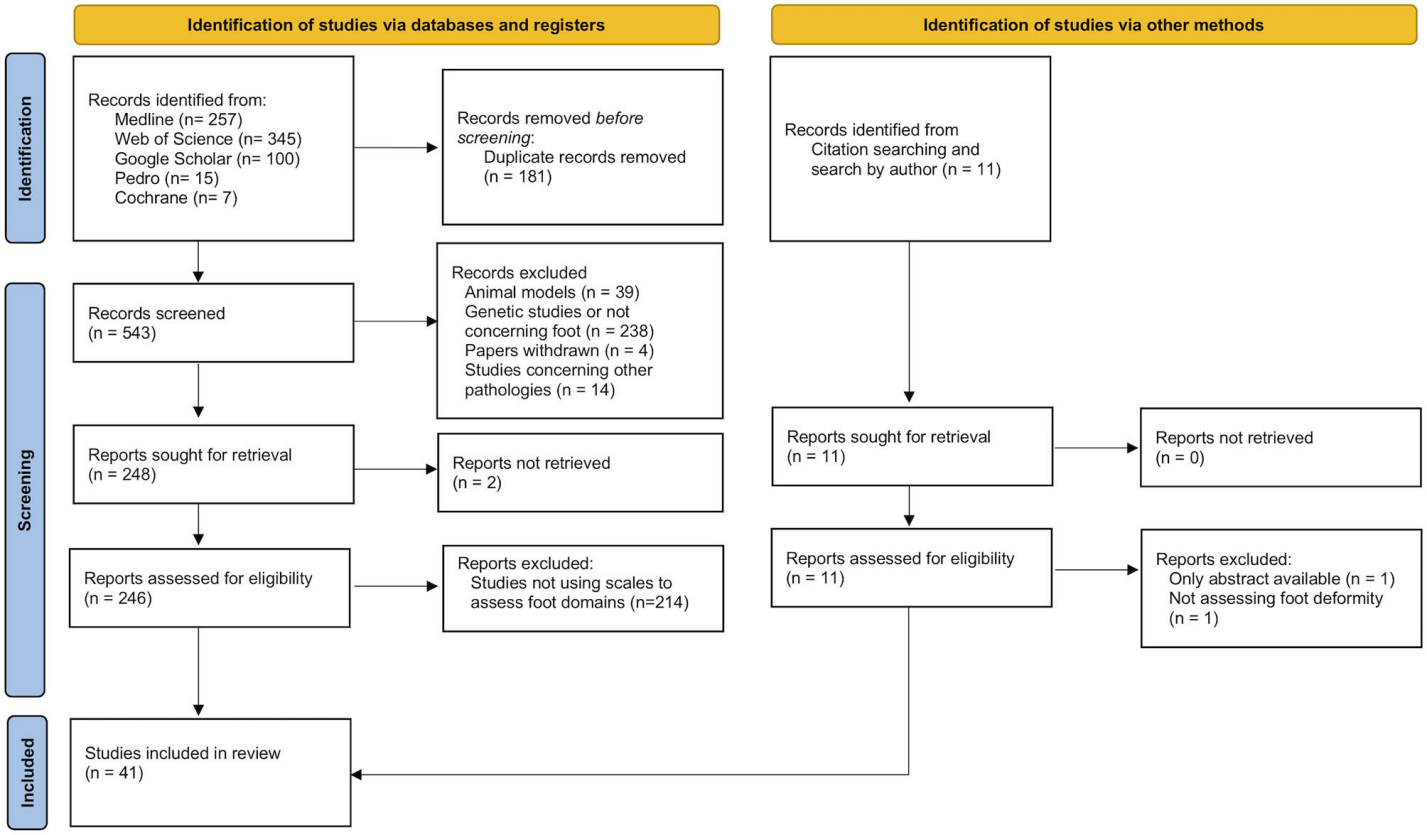
Flow diagram of the selection process of papers using clinical scales to assess foot deformity in CMT patients.

The first step of the search found 724 papers. Of these, 41 were included in the review because they met the aim of this research. Of the 683 excluded studies, only three required a discussion between both investigators. The selection procedure is presented in Figure 1. The selected studies used six different scales: “Foot Posture Index” (FPI), “Foot Function Index” (FFI), “Maryland Foot Score” (MFS), “American Orthopedic Foot & Ankle Society's Hindfoot Evaluation Scale” (AOFAS-AHES), “Foot Health Status Questionnaire” (FHSQ), and “Wicart-Seringe grade” (WSG).

All included papers are listed in Table 1 and grouped by the foot assessment scale used. The main characteristics of each scale are also summarized in Table 1. Papers in which more than one tool was used were included more than once.

**Table 1:** Scales used in literature for foot assessment in CMT patients, related articles, and main characteristics.

**Scale name**	**Acronym**	**References**	**Description**	**Scoring system**	**Range**	**Delivered / self-assessment**
Foot Posture Index, 6-item version	FPI-6	Burns et al. (2005) Crosbie and Burns (2007) Keenan et al. (2007) Crosbie et al. (2008) Burns et al. (2009) Rose et al. (2010a) Rose et al. (2010b) Burns et al. (2010) Blyton et al. (2011) Pagliano et al. (2011) Burns et al. (2012a) Rose et al. (2015) Cornett et al. (2016) Kennedy et al. (2016) Wegener et al. (2016) Wojciechowski et al. (2017) Kennedy et al. (2017) Kennedy et al. (2018) Stino et al. (2019) Baptista et al. (2021) Kennedy et al. (2019) Lin et al. (2019) Estilow et al. (2019) Bray et al. (2020) Pogemiller et al. (2020) Ramdharry et al. (2021)	6 items based on the observational analysis of the hindfoot and the rearfoot	Each item has a score comprised between −2 and 2 on a Likert scale. The score depends on the clinical evaluation of foot alignment excluding external factors.	−12 to 12 from supinated or cavovarus features (−12 to −1), to neutral (0 to +5) to pronated or planovalgus features (+6 to +12). A conversion of the ordinal total score into a numeric value has been provided by Keenan and Colleagues (range −10.47 to 8.65). It is advisable to use this converted scoring system.	Delivered
Foot function index	FFI	Ward et al. (2008) Leeuwesteijn et al. (2010) Bihel et al. (2019) Klerken et al. (2019)	23 items grouped into three subscales dealing with activity limitation, disability, and pain.	The item's score is included between 0 and 10 and rated using a visual analog scale (VAS). The subscales scores are averaged to obtain a total mean score.	Range: 0–100. The highest FFI score represents the lower level of function.	Self-assessment
Maryland Foot Score	MFS	Faldini et al. (2015)	6 items to estimate pain and function of the foot and ankle complex.	Different items are characterized by different weighted scores.	Range: 5–100. The highest score represents the best condition.	Partially delivered and partially self-assessment
American Orthopedic Foot & Ankle Society's Hindfoot Evaluation Scale	AOFAS-AHES	Kołodziej et al. (2013) Gordon et al. (2013) Dreher et al. (2014) Napiontek and Pietrzak (2015) Ettinger et al. (2016) Simon et al. (2019) Ergun and Yildirim (2019) Klerken et al. (2019) Alammar et al. (2020)	10 items dealing with pain, function, and alignment	Different items are characterized by different weighted scores	Range: 0–100. The highest score represents the best condition. Subscales' ranges: pain (0–40), function (0-50), alignment (0–10).	Partially delivered and partially self-assessment
Foot Health Status Questionnaire	FHSQ	Crosbie et al. (2008)	13 items centered on pain, function, footwear and general foot health	Each item is scored using a Likert scale.	Range: 0–100. The highest score represents the best condition.	Self-assessment
Wicart-Seringe grade	WSG	Wicart and Seringe (2006) d'Astorg et al. (2016) Simon et al. (2019)	Grading based on a combination of the talar valgus (y/n), neutral heel (y/n), talar varus (y/n) and of the Méary angle	A single grading is provided, on four levels	Range: Poor–Very Good	Delivered

FPI was developed to evaluate the overall foot position considering its three-dimensional nature (Redmond et al., 2006). A comprehensive review of the literature surrounding the clinical evaluation of foot was performed, leading to a list of 36 items. According to the best practices for item selection when developing and validating clinical scales (Boateng et al., 2018), this list was narrowed down to eight items (FPI-8) (Evans et al., 2003; Scharfbillig et al., 2004; Redmond et al., 2006; Keenan et al., 2007). Next, two items not belonging to the *foot function* domain were also removed, leading to the final FPI-6 version (Redmond et al., 2006). Twenty-six papers used FPI to assess CMT patients in different settings in both the adult and pediatric populations (Burns et al., 2005, 2009, 2010, 2012a; Crosbie and Burns, 2007; Keenan et al., 2007; Crosbie et al., 2008; Rose et al., 2010a,b, 2015; Blyton et al., 2011; Pagliano et al., 2011; Cornett et al., 2016; Kennedy et al., 2016, 2017, 2018, 2019; Wegener et al., 2016; Wojciechowski et al., 2017; Estilow et al., 2019; Lin et al., 2019; Stino et al., 2019; Bray et al., 2020; Pogemiller et al., 2020; Baptista et al., 2021; Ramdharry et al., 2021).

FFI was developed in 1991 to assess foot function in patients with rheumatoid arthritis, without fixed foot deformities or prior foot surgery (Budiman-Mak et al., 1991). FFI metric properties have been assessed mainly in patients with orthopedic foot pathologies (Budiman-Mak et al., 1991; Saag et al., 1996; Kuyvenhoven et al., 2002; Agel et al., 2005; Madeley et al., 2012; Pinsker et al., 2015; Bihel et al., 2019). Four papers used FFI in the evaluation of CMT patients (Ward et al., 2008; Leeuwesteijn et al., 2010; Bihel et al., 2019; Klerken et al., 2019). Of these, one focused on the metric properties of FFI when used with patients with type 1A CMT disease.

AOFAS-AHES and MFS were developed for trauma and orthopedic patients, and their scores merged aspects of alignment, pain, and loss of activity (Heffernan et al., 2000; SooHoo et al., 2003; Ibrahim et al., 2007; Pena et al., 2007; Schepers et al., 2008; Madeley et al., 2012; Cöster et al., 2014; Pinsker et al., 2015; Conceição et al., 2016; Ponkilainen et al., 2020). In these tools the walking impairment is presumed to be a direct consequence of an acute foot pathology. FHSQ is a self-administered questionnaire developed and validated to measure the quality of life related to foot health in a population suffering from minor foot conditions, such as skin and nail disorders (Bennett et al., 1998). AOFAS-AHES was used in nine orthopedic studies to evaluate a cohort of CMT patients (Gordon et al., 2013; Kołodziej et al., 2013; Dreher et al., 2014; Napiontek and Pietrzak, 2015; Ettinger et al., 2016; Ergun and Yildirim, 2019; Klerken et al., 2019; Simon et al., 2019; Alammar et al., 2020) while MFS and FHSQ were both employed only once (Crosbie et al., 2008; Faldini et al., 2015).

WGS was used for the first time in a study by Simon and colleagues in 2019 (Simon et al., 2019) to assess the effects of osteotomy surgery in children with cavo-varus deformity. WGS was then used in another two studies conducted by the same research team (Wicart and Seringe, 2006; d'Astorg et al., 2016).

### Papers Addressing the Metric Properties of the Scales

**Figure 2:**
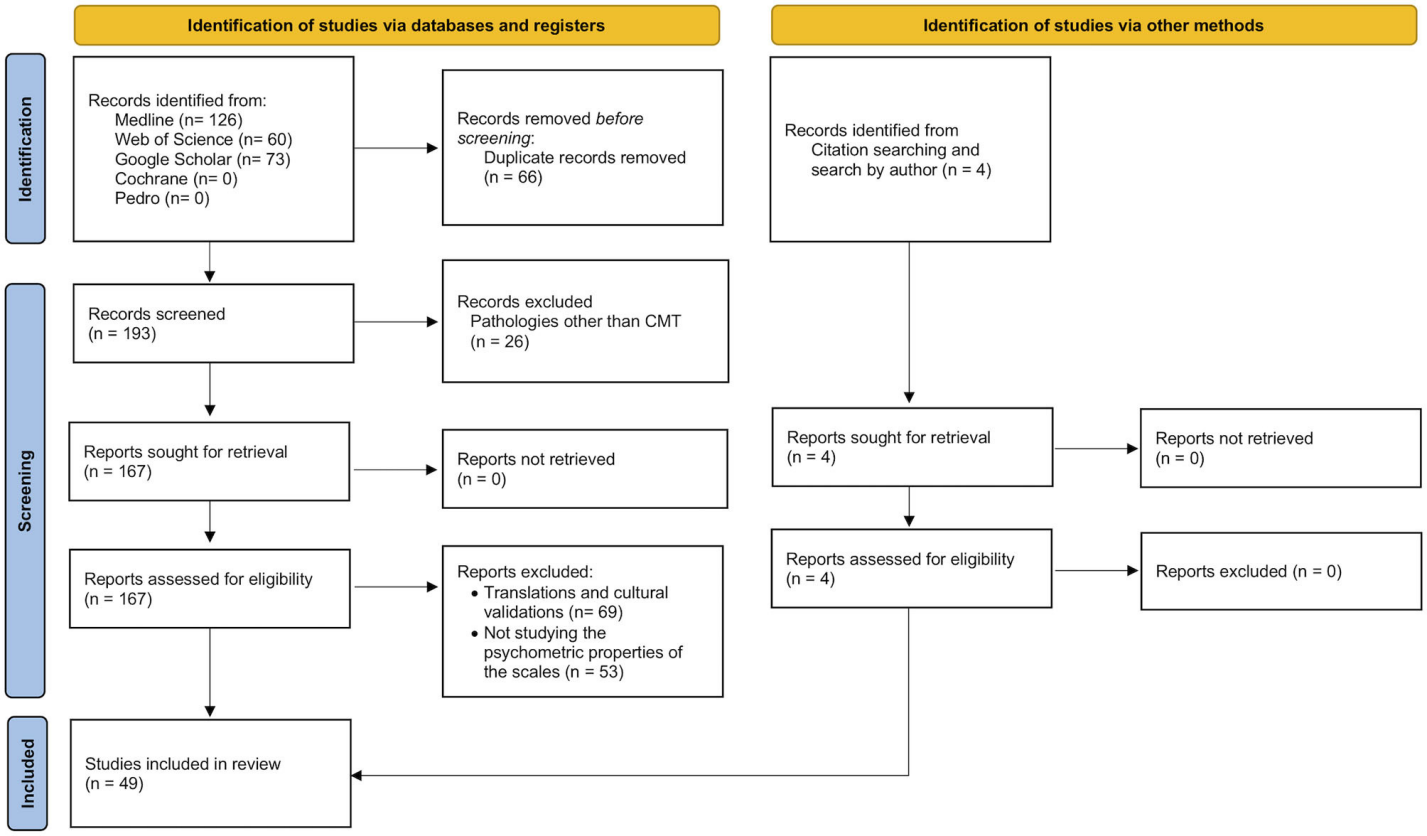
Flow diagram of the selection process for studies assessing the psychometric properties of the scales identified previously.

As detailed in the method section, a further analysis of their psychometric properties was conducted for each of the scales found in the previous search and selection. This second search initially produced 259 papers. After the removal of duplicates, 148 further papers were excluded because they did not add any extra information about the metric properties of the tools and/or because of their application in pathologies very different from CMT, resulting in 45 primary studies. Four more primary studies were added based on the bibliographic references of two other reviews (Evans and Rome, 2011; Budiman-Mak et al., 2013), leading to a total of 49 primary studies. The selection procedure is presented in Figure 2.

The metric properties of the scales have been collected in terms of number of raters and their expertise, indicators of internal consistency (e.g., Cronbach's alpha), intra-rater and inter-rater reliability (e.g. ICC), and of concurrent validity (e.g. r, R^2^). These are listed in Table 2.

**Table 2:** Sample characteristics and metric properties of the scales as reported in all studies included.

**Scale listed by Author, Year**	**Sample**	**Metric properties**
**Foot Posture Index – 8 items (FPI-8)**		
Evans et al. (2003)	58 feet/ 29 healthy children (4–6 y) 60 feet/ 30 adolescents (8–15 y) 60 feet/ 30 adults (20–50 y)	No. of raters: three for children; four for adolescents; four for adults. Inter-rater reliability: ICC = 0.62 in children, ICC = 0.74 in adolescents and ICC = 0.58 in adults
Scharfbillig et al. (2004)	31 healthy adults with pronate, normal and supinate foot age: > 40 y	Concurrent validity study 4 FPI subdomain scores v. four corresponding angles obtained from radiographic images. Only one correlation found. The analysis suffered from methodological issues
Keenan et al. (2007)	143 participants; 131 healthy and 12 CMT adults age range 8-65 y	No. of raters: not reported. Rasch analysis. Misfit to the Rasch model Lack of uni-dimensionality. (*x*^2^ test = 27.63, *p* = 0.03), with two items to be removed- Absence of differential item functioning for all FPI-8 items. Good person separation index (PSI 0.88)
**Foot Posture Index – 6 items (FPI-6)**		
Oleksy et al. (2010)	60 healthy children age range 9–16 y	No. of raters: not reported. Test-retest; r = 0.89–0.96
Menz (2006)	95 healthy elderly age range 62-94 y	Intra-rater ICC = 0.27–0.81 Concurrent Validity: FPI v. radiographic measures of static foot posture (calcaneal first metatarsal angle, calcaneal inclination, navicular height, navicular height from XRay; normalized navicular height, normalized navicular height from radiograph; normalized navicular height from radiograph (truncated), normalized navicular height (truncated), 0.360 ≤ |r| ≤ 0.593, *p* <0.01
Redmond et al. (2006)	131 club athletes age 18–65 y	Cronbach's alpha >0.85 Concurrent validity vs. a set of angles obtained by 3F kinematics (20 healthy adults. 21–45 y). The relevant variables from the motion tracking lower limb model predicted 58–80% of the variance in the six FPI components.
Redmond et al. (2006)	14 healthy adults age range 18–57 y	Concurrent validity with the ankle joint angles assessed by 3D kinematics in static conditions in neutral, forced inverted and forced everted position: The FPI-6 scores predicted 64% of the variation in the static inversion-eversion angle (adjusted *R*^2^ = 0.64, *F* = 73.529, *p* <0.001) FPI predictive ability of ankle joint mobility during walking: FPI total score predicted 41% of the dynamic variation in midstance foot position.
Keenan et al. (2007)	143 participants: 131 healthy and 12 CMT adults age range 8–65 y	No. of raters: not reported. Good overall fit to the Rasch model (*x*^2^ test = 11.49, *p* = 0.49). One-dimensionality. Absence of differential item functioning for all FPI-6 items. Good Person-separation index (PSI=0.884). Conversion of the ordinal raw score into numerical values, based on a larger dataset of data including *n =* 426 subjects
Cornwall et al. (2008)	92 feet from 46 healthy adults mean age 26.0 ± 4.8 y	No. of raters: three with different expertise Intra-rater reliability: ICC = 0.928–0.937 Inter-rater reliability ICC = 0.525–0.655 Learning curve: at least 20 assessments recommended before using the tool
Crosbie et al. (2008)	16 patients with CMT age range 31-82	The association between pain FPI score and foot pressure patterns as assessed by in-shoe systems was investigated. An association was found between forefoot and midfoot pressure values and the FPI score.
Morrison and Ferrari (2009)	30 healthy children and teenagers age range 5 – 16 y	No. of raters: 2. These were experienced podiatrists, trained on FPI before participating in the study. Inter-rater reliability: weighted Cohen's K on FPI-6 score Kw= 0.86 (CI not reported). Cohen's K = 0.57 (computed based on data reported in this Table). weighted Cohen's K on foot classification Kw = 0.88 (CI not reported).
Evans et al. (2012)	30 healthy children age range 7–15	No. of raters: 2. One experienced and one newly graduated podiatrist. Two assessments each, separated by at least two hours Intra-rater reliability: ICC = 0.86–0.97 Inter-rater reliability: ICC = 0.38 - 0.74 at the first assessment, ICC = 0.86 – 0.97 at thee second assessment. SEM <2
Griffiths and McEwan (2012)	26 healthy adults mean age 25.9 ± 9.2 y	No. of raters: 2 with different expertise Intra-rater reliability: ICC = 0.412 Inter-rater reliability: ICC = 0.143
Terada et al. (2014)	40 healthy adults, both feet assessed mean age 23.7 ± 8.5 y	No. of raters: 2. These were certified athletic trainers with no previous experience using the FPI-6 and trained on 15 subjects. Picture-based assessment. Three assessments separated by a day. Intra-rater reliability: ICC_left = 0.925–0.975, ICC_right = 0.931–0.977. Insufficient inter-rater reliability: ICC <0.5, SEM= 3. Cohen's k_left=0.12 and k_right= 0.19.
Evans and Karimi (2015)	728 children age range 3–15 y	No association between body mass and flatfeet in children: r = −0.077, P <0.05.
Tucker et al. (2015)	46 children (normal-weight and obese) 10.5 ± 1.4 y	No of raters: 3 trained physiotherapists Intra-rater reliability: ICC = 0.979 (0.966–0.988), 0.989 (0.982–0.994) Inter-rater reliability: ICC = 0.788 (0.597–0.887) for nonobese. ICC = 0.834 (0.735–0.901) for obese children.
McLaughlin et al. (2016)	83 healthy adults age not reported	No. of raters: 2, unexperienced. Rasch-converted total FPI-6 score. Inter-rater reliability ICC_left = 0.80 – 0.91, ICC_right = 0.78 – 0.90 Level of agreement on foot type classification: 63/83 (76%) for the left foot and 68/ 83 (82%) for the right foot; Cohen's Kappa: 0.60 – 0.86 and 0.59 – 0.86, respectively.
Gijon-Nogueron et al. (2016)	1762 healthy children age range 6-11 y	No. of raters: 2. These were experienced podiatrists Reference values in childhood. Median value: FPI =4, except for the right foot among girls (FPI=3). 85th percentile: FPI=6, uniformly among subjects. This is considered to represent the boundary between the normal and the pronated foot among children. Mean values: FPI_right = 3.74 (SD 2.93); FPI_left = 3.83 (SD 2.92)
Aquino et al. (2018)	21 healthy adults mean age 27 ± 10 y; 19 older adults mean age 73.5 ± 8 y;	No. of raters: 2. These were 1 experienced PT and 1 PT student; 2 assessments separated by 7–15 days Intra-rater reliability: ICC = 0.66 (0.45–0.80), for adults. ICC = 0.41 (0.11–0.64), for older adults. Inter-rater reliability: Cohen's κ = 0.47–0.56, for adults. Cohen's κ = 0.40-0.48, for older adults.
Kenny et al. (2018)	38 healthy dancers age range 16.6–19.2	No of raters: 9 trained physiotherapists and kinesiology graduate students Test-retest reliability: ICC = 0.75 (0.56-0.86), for left foot. ICC = 0.63 (0.39-0.79) per right foot.
Zuil-Escobar et al. (2019)	71 young adults with low medial longitudinal arch mean age 24.1 ± 3.4 y	No. of raters: 2. These were experienced PTs; 2 assessments separated by 48 hours Intra-rater reliability: Cohen's κ = 0.872 (*n* = 20) Inter-rater reliability Cohen's κ = 0.829 (*n* = 20) Concurrent Validity: FPI-6 v. navicular drop test: r = 0.818, P <0.001 FPI-6 v footprint parameters: *r* = |0.663–0.703|, *P* <0.001
Hegazy et al. (2020)	612 children, 1224 feet age range 6-18 y	No. of raters: 1 physiotherapist with 12 years of expertise Intra-rater reliability: ICC = 0.96, P <0.001 Diagnostic accuracy: AUC = 0.82 (0.78-0.85)
Patel et al. (2020)	33 healthy adults, 66 feet age range 18-79 y	No. of raters: 2. Intra-rater reliability: ICC = 0.982-0.993 for rater 1 and ICC=0.905-0.963 for rater 2 Inter-rater reliability: ICC = 0.593-0.759 Concurrent Validity with 3D angles computed from 3D tomography: FPI v. Foot and Ankle Offset r = 0.794, p <0.001; FPI v. Calcaneal Offset r = 0.781, p <0.001; FPI v Hindfoot Alignment Angle r = 0.80, p <0.001. Subgroup analysis revealed the strength of association dropped when the hindfoot had a valgus alignment.
Kirmizi et al. (2020)	60 healthy young adults age range 18–40 y	No. of raters: 2. These were experienced PTs; 2 assessments separated by 48 hours Intra-rater ICC: 0.910–0.967 (*n =* 60) Inter-rater ICC: 0.281–0.771 (*n =* 30), SEM ≤ 2
**Foot Posture Index - 5 points version (FPI-5)**		
Kuyvenhoven et al. (2002)	206, Non traumatic foot or ankle problem, age ≥ 45 years	internal consistency: Cronbach's alpha = 0.93 (IC not reported). Inter-assessor ICC 0.76 – 0.85
**Foot Function Index (FFI)**		
Budiman-Mak et al. (1991)	87 patients with rheumatoid arthritis age range 24–79 y	Internal consistency: Cronbach's alpha 0.956 (CI not reported) for the total score, with the lowest consistency values in the activity limitation subscale (α=0.733) and the highest consistency for the pain subscale (0.956). Factor analysis grouped items in 4 domains. Items belonging to the pain and disability subscales were properly grouped in two separated items. Conversely, items belonging to the activity limitation subscale were grouped in two factors, related to limitations and to the use of assistive devices.
Saag et al. (1996)	30 patients with rheumatoid arthritis, mean age 57.5 ± 11.6 y	Pain subscale only was analyzed, referred to as FFI VAS. 86% of subjects correctly completed the assessment; Internal consistency: Cronbach's alpha > 0.92 (among left, right, assessment1, assessment2). Test-retest ICC_right = 0.79–0.95, ICC_left = 0.74–0.93
Agel et al. (2005)	54 subjects with forefoot complaints or hindfoot/ankle complaints or deformity mean age 51 years (SD not reported)	Scores arbitrarily assessed on a Likert scale instead of a 0-9 VAS Test-retest analysis for single items: the percentage of patients with (item_score_2 item_score_1) = 0 ranged between 23% and 79% among items.
Baumhauer et al. (2006)	11 patients with rheumatoid arthritis age range 40-72 y	Test-retest ICC = 0.85
SooHoo et al. (2006)	25 subjects who underwent foot surgery due to chronic condition age range 21–69 y	Responsiveness: SRM = −0.39 and ES−0.55 for the Activity Limitation domain; SRM−0.83 and ES−0.86 for Pain, and SRM−0.68 and ES−0.75 for Disability.
SooHoo et al. (2006) (2)	69 subjects with a chronic condition affecting the foot and ankle age range 16–82y	Concurrent validity (compared to SF-36 items): r =-0.32 -−0.69 (P <0.05), disability domain; r = −0.28 -−0.64 (P <0.05), activity limitation domain, r = −0.32 -−0.69 (P <0.05), pain domain.
Madeley et al. (2012)	117 patients who underwent ankle replacement or arthrodesis mean age 59.7 y (27–84)	Concurrent validity (compared to SF-36): *r* = 0.61, *P* <0.0001 Responsiveness: SRM = 1.04 and ES 1.37
Pinsker et al. (2015)	142 post-operative patients with end-stage ankle arthritis mean age 61.2 y (22–92)	Test-retest reliability: ICC = 0.93 Internal consistency: Cronbach's alpha = 0.96
Muradin and van der Heide (2016)	30 subjects with Rheumatoid Arthritis age range 44–76 y	SRM = −0.85; SES = −0.80; GRR = −1.25
Bihel et al. (2019)	26 patients with type 1A Charcot-Marie-Tooth disease age range 29-83 y	Internal consistency: Cronbach's alpha = 0.95 (IC95% not reported) Inter-rater reliability: Lin's concordance coefficient 0.73–0.98 External consistency: FFI v. SF-36 physical composite score correlation (*r* = −0.58 *P* <0.005), FFI v. gait cadence (*r* = −0.52; *P* <0.05); FFI was di not correlate with other kinematics- and kinetic-related parameters of gait.
**Maryland Foot Score (MFS)**		
Heffernan et al. (2000)	25 subjects who underwent to calcaneal fractures' internal fixation age range 22–65 y	Concurrent Validity MFS pain v. SF-36 pain: *r* = 0.64, *p* <0.001; MFS physical function v. SF-36 physical function: *r* = 0.78, *p* <0.001
Schepers et al. (2008)	48 postoperative patients with calcaneal fractures, 59 feet Median age 49 ± 13 y	Internal consistency: Cronbach's alpha = 0.82 Spearman rank test (correlation between MFS and AOFAS): *rho* = 0.84, *P* <0.001
**American Orthopedic Foot and Ankle Society (AOFAS) scale**		
SooHoo et al. (2003)	91 patients with foot or ankle pathologies; mean range 50 y, (SD not reported)	Poor relation with SF-36 sub-scales in the overall study population (Pearson correlation coefficients 0.02 to −0.36). Higher correlation for the patients with ankle-hindfoot disorders (0.11 to 0.53) rather than patients with forefoot disorders (−0.05 to 0.25).
SooHoo et al. (2006)	25 subjects who underwent foot surgery due to chronic condition age range 21–69 y	Responsiveness: SRM = 1.10; ES = 1.12
Pena et al. (2007)	154 End stage ankle arthritis patients, undergoing total ankle replacement age not reported	AOFAS v. Musculoskeletal Function Assessment (MFA); patients assessed preoperatively, and at 6, 12, and 24 months after surgery. At the 1-year mark, mild significant correlations (|rho| ranging between 0.27 and 0.65) was found between AOFAS and MFA pain-related items, function-related items, and total scores.
Ibrahim et al. (2007)	45 Patients awaiting foot surgery, age range 21-66 years	Concurrent Validity: moderate correlation with FFI (|r| = 0.68) Test-retest: non significative group difference; ICC and/or SEM not computed. Responsiveness: significative group difference between per and post-surgical values
Schepers et al. (2008)	48 postoperative patients with calcaneal fractures, 59 feet Median age 49 ± 13 y	Internal consistency: Cronbach's alpha = 0.78 Spearman rank test (correlation between AOFAS and MFS): *r* = 0.84, *P* <0.001
Dawson et al. (2012)	262 patients who underwent foot/ankle surgery mean age 53 y	Responsiveness: ES = 1.29
Madeley et al. (2012)	117 patients who underwent ankle replacement or arthrodesis mean age 59.7 y (27-84)	Concurrent validity (compared to SF-36): *r* = 0.61, *P* <0.0001 Responsiveness: SRM = 1.34; ES 1.69
Cöster et al. (2014)	206 patients with great toe or ankle/hindfoot disorders median age 56 y (24-81)	No of raters: not specified; trained physiotherapists Inter-rater reliability: ICC = 0.70 for great toe disorders group, ICC = 0.81 for ankle/hindfoot disorders group. Internal consistency: Cronbach's alpha = 0.15 for great toe disorders group, Cronbach's alpha = 0.42 for ankle/hindfoot disorders group Responsiveness: Effect size = 1.05 for great toe disorders group, Effect size = 1.73 for ankle/hindfoot disorders group.
Pinsker et al. (2015)	142 post-operative patients with end-stage ankle arthritis mean age 61.2 y (22-92)	Test-retest reliability: ICC = 0.89 Internal consistency: Cronbach's alpha = 0.84
Conceição et al. (2016)	33 female patients with rheumatoid arthritis mean age 53 ± 10.9 y	Intra-rater reliability: ICC = 0.95, *P* <0.001 Inter-rater reliability: ICC = 0.91, *P* <0.001 Rasch analysis: 8 items were satisfactory, 1 was identified as erroneous
Ponkilainen et al. (2020)	117 patients with Lisfranc injuries mean age 41 ± 17 y	Internal consistency: Cronbach's alpha = 0.75 Convergent validity (compared to Visual Analog Scale – Foot and Ankle): r = 0.89
**Foot-Health Status Questionnaire (FHSQ)**		
Bennett et al. (1998)	111 subjects with “Skin, nail and musculoskeletal condition” mean age 54 ± 20 y	Internal consistency: Cronbach's alpha = 0.85–0.88 Intra-rater reliability (*n =* 72): ICC = 0.74–0.92. Lowest ICCs are in footwear domain (ICC=0.740) and general foot health domain (ICC = 0.784); confidence intervals not reported.
Landorf and Keenan (2002)	17 subjects with plantar fasciitis mean age 45 ± 10 y	Concurrent Validity study: FHSQ and FFI were completed before and at 4 weeks after receiving foot orthotics. The results of the study demonstrated that the changes in the FHSQ scores were greater than the changes in the FFI scores. The FHSQ score significantly improved after treatment, while FFI did not.
Crosbie et al. (2008)	16 patients with CMT age range 31-82	The association between pain (first item of the FHSQ score) and foot pressure patterns as assessed by in-shoe systems was investigated. No association was found.
Cuesta-Vargas et al. (2012)	22 healthy elderly mean age 66.8 ± 7.6 y	Concurrent Validity study: FHSQ v. clinical and functional variables, measures of foot strength and plantar pressure: 0.4 < |r| <0.5, *p* <0.05.
Menz et al. (2014)	59 older adults with foot pain mean age 82.3 ± 7.8 y mean age	Responsiveness: SRM = −0.50 and Cohen's d = 0.63 for the pain domain; SRM = −0.26 and Cohen's d = 0.37 for the function domain; SRM = −0.12 and Cohen's d = 0.09 for the footwear domain; SRM = −0.27 and Cohen's d = 0.29 for the foot health domain.
**Wicart-Seringe grade**		No validation studies found.

*ICC, Intraclass Correlation Coefficient; r, Pearson's correlation coefficient; rho, Spearman's correlation coefficient; R^2^, coefficient of determination; SRM, standardized response mean; ES, effect size; SES, standardized effect size; GRR, Guyatt responsiveness ratio. PSI, person separation index*.

Several versions of FPI were developed and analyzed before defining which was best, following an iterative process typical in the construction of assessment scales (Kuyvenhoven et al., 2002; Evans et al., 2003; Scharfbillig et al., 2004; Keenan et al., 2007). FPI-8 has been used in the early 2000s and was tested by three authors in samples of both healthy subjects and CMT patients aged from 4 to 65. It showed medium inter-rater reliability (ICC: 0.58–0.74) and a lack of uni-dimensionality. After the removal of two items not linked to the foot deviation domain, the metric properties of the new FPI-6 were analyzed in 21 studies from 2006 to 2020. Study samples ranged from 14 to 1762 healthy adults and children. Only one study considered 12 CMT adults from an assessed sample of 143 participants (Keenan et al., 2007). The scale showed an appropriate internal consistency (Cronbach's alpha > 0.85) (Redmond et al., 2008), and the Rasch analysis by Keenan et al. confirmed its uni-dimensionality. Moreover, the scale was found to be well suited to be used at the single-patient level, because of a Person Separation Index > 0.85 (Keenan et al., 2007). Finally, the Rasch procedure converts the ordinal FPI-6 score to a numerical value, which is proportional to the amount of foot deviation (Keenan et al., 2007). Inter-rater reliability was 0.59 – 0.97 when the scale was administered by expert assessors. Raters in the included studies were podiatrists, athletic trainers, physiotherapists, and kinesiologists. Concurrent validity was demonstrated by comparing the FPI-6 score to Xray and tomography related measures, 3D kinematics, and other clinical measurements such as the navicular drop test. No study addressed the responsiveness of the scale, yet.

The metric properties of FFI have been assessed in ten studies, dated 1991–2019. Samples usually included patients with rheumatoid arthritis, foot deformities or who underwent arthrodesis, with sample sizes ranging from 11 to 142 individuals. Authors found high internal consistency and inter-rater reliability (ICC: 0.74 – 0.95). Bihel and colleagues investigated the metric properties of FFI in 26 CMT patients, and found excellent internal validity (Cronbach's α = 0.95) and satisfactory reproducibility (Lin's concordance coefficient = 0.82) (Bihel et al., 2019). Test-retest ICC was in the order of 0.8-0.9 (Saag et al., 1996; Baumhauer et al., 2006). However, FFI activity subscale demonstrated low external validity when compared with gait patterns. Adequate responsiveness of the scale to the variations determined by surgery was found in the three studies addressing this topic (SooHoo et al., 2006; Madeley et al., 2012; Muradin and van der Heide, 2016).

Two studies tested MFS in a population of 25–48 patients who suffered from calcaneal fracture. Schepers and colleagues found high internal consistency (Cronbach's alpha = 0.82) (Schepers et al., 2008), but moderate concurrent validity when the scale was compared to SF-36 (Heffernan et al., 2000). Information on the raters characteristics were not available.

Eleven authors investigated the metric properties of the AOFAS from 2003 to 2019. Samples ranged from 25 to 262 patients suffering from diverse orthopedic or rheumatic pathologies. Among authors, only Cöster specified that trained physiotherapists administered the test (Cöster et al., 2014). Internal consistency was different among studies, ranging from 0.15 to 0.78. Authors found low to moderate concurrent validity with other clinical measures, such as the Visual Analogical Scale, the SF-36, or the Musculoskeletal Functional Assessment, and moderate inter-rater reliability (ICC = 0.70–0.91). Responsiveness was satisfactory (see Table 2), as found in three studies on foot surgery involving large samples (SooHoo et al., 2006; Dawson et al., 2012; Madeley et al., 2012), also thanks to the large effect of surgery on foot alignment and pain.

FHSQ was investigated in five studies from 1998 to 2014. Bennet and colleagues found high internal consistency (Cronbach's alpha = 0.85 – 0.88). The overall foot condition in patients with plantar fasciitis, or secondary skin and nail issues was assessed, and FHSQ was found to have moderate to good reliability (Bennett et al., 1998) and moderate concurrent validity with a set of clinical and functional variables and with measures of foot strength and plantar pressure (Landorf and Keenan, 2002; Cuesta-Vargas et al., 2012). Crosbie adopted FHSQ in a cohort of CMT patients with cavus foot deformity and found no relationship between the FHSQ score and the amount of cavus deformity assessed with sensors for plantar pressure and foot-ground contact duration (Crosbie et al., 2008). Inadequate responsiveness of the whole tool was also reported by Menz and colleagues in a study on the effect of specific footwear on foot status in older adults with persistent foot pain (Menz et al., 2014). While the subscales assessing pain and function detected improvements, the remaining subscales on footwear and general foot health did not, leading to a low global responsiveness.

Our search did not find any studies investigating the metric properties of WSG.

## Discussion

The aim of this study was to provide an overall view of the clinical scales used to assess the foot in CMT patients, and help clinicians choose the best scale to employ in their daily practice. For this reason, we conducted a scoping review collecting all clinical scales used so far in literature, describing the scales' development and metric properties. Scoping reviews are better suited, as they do not aim at answering a specific question—as systematic reviews do—but aim at mapping existing evidence and analyzing any gap in knowledge (Tricco et al., 2018; Peters et al., 2020).

We found 42 studies using six different scales for foot assessment in the CMT population (Table 1) and 49 studies assessing their metric properties (Table 2). Their history, internal consistency, inter-rate and intra-rate reliability, and assessing modalities are summarized in Table 2.

### Critical Appraisal of the Scales Used for CMT Foot Assessment

The literature search revealed two different types of scales: those built specifically for neurologic foot assessment, including CMT patients, and those borrowed from the orthopedic or rheumatologic fields and then used to assess the neuropathic foot.

FPI-6 is the only scale specifically developed for CMT patients being the most widely used scale assessing foot deformity and was employed in 27 studies included in the current review. Its broad use is mainly due to its uni-dimensionality (i.e., the power to address a single construct) and satisfactory psychometric properties (e.g., inter-rater reliability). This version is a product of fine-tuning previous versions (Martin and Irrgang, 2007) and following the criteria for the creation of a new assessment scale.

Other scales such as FFI, MFS, AOFAS-AHES, FHSQ, and WSG have poor psychometric properties when used with neurologic patients (see Table 2). This might be traced back some missteps during their set up. In fact, unlike FPI, these scales were developed and evaluated only for orthopedic or rheumatologic patient cohorts. For this reason, the assessment of foot deviation provided by these scales probably does not include all the aspects that should be considered when dealing with more neurologically complex patients. Moreover, these scales were mainly developed during clinical practice and did not undergo all the steps necessary to build a new measurement scale (Boateng et al., 2018).

FFI was developed for rheumatic patients and not all domains have proven to possess good external validity, such as the activity subscale in patients with rheumatoid arthritis. Responsiveness of this scale has been demonstrated and this supports the use of FFI to monitor outcomes in patients with orthopedic conditions. However, this subscale is reasonably linked to several factors other than foot deviation consequent to peripheral neuropathy. Another issue of this tool relies on the exclusion of subjects with fixed foot deformities during its validation, while 71% of CMT patients present this kind of foot deformity (Saag et al., 1996; Stino et al., 2019). Hence, its use with CMT patients remains questionable.

AOFAS-AHES and MFS were mainly developed for orthopedic patients with specific issues caused by an acute ankle-foot injury. Responsiveness was satisfactory, also thanks to the large effect of surgery on foot alignment and pain. However, when dealing with CMT patients, many factors must be considered when assessing walking impairment, such as muscle atrophy and weakness, sensory deficiency, and foot deformity. Therefore, scales designed for orthopedic patients should not be used with neurologic patients.

Finally, FHSQ was developed to test patients with plantar fasciitis or non-serious skin pathologies. When used with CMT, no correlation was found between its score and the percentage of cavus deformity (Crosbie et al., 2008; Cuesta-Vargas et al., 2012). Although the subscales assessing pain and function were found to have good responsiveness, the remaining subscales did not. This is a common drawback in tools assessing multiple domains. The use of this tool in a cohort of patients should be adopted with caution, as mentioned by Landorf and colleagues (Landorf and Keenan, 2002), who suggested limiting the use of FHSQ for pathologies where walking ability is not compromised.

Most of the scales considered in this scoping review (FFI, MFS, AOFAS and FHQS) focus on a general assessment of the whole lower limb function, investigating pain, perception of stability and limping, difficulty while performing ADLs (walking indoors or outdoors, climbing stairs, getting up from a chair, stepping over an obstacle), and use of appropriate walking aids and shoes (see Table 1). The AOFAS includes a subscale specific for foot alignment, while FPI and WGS focus on the single domain of foot posture. When focusing on foot deformity and on the effect of any corrective interventions, unidimensional scales assessing foot posture should be used. At the same time, from an ICF classification perspective, the impact of foot posture and pain on functional activities should also be assessed. In line with the aims of this scoping review and for the above-mentioned reasons, we suggest the use of FPI-6 when assessing foot deformities in CMT patients.

### Focusing on FPI-6: Current Strengths and Incentives for Increased Future Use

Currently, FPI-6 is the most appropriate tool to be used for foot assessment in CMT patients. The FPI-6 is scale involving six items related to rearfoot and forefoot components, used to quantify the degrees of foot pronation or supination while standing. It investigates the position of the talar head, the calcanear inversion/eversion, the lateral malleolus, the talo-navicular congruence, the medial arch height, and the forefoot abduction/adduction. Each item in scored on between−2 to +2, and the item scores are summed up to obtain a global score. A positive final score > 5 points reveals a pronated foot, a negative final score suggests a supinated foot, while a score of 0–5 indicates a neutral foot position (Redmond et al., 2006, 2008). This was specifically designed to assess foot deformities in neurological patients and proved to be sensitive to disease-related postural changes (Scharfbillig et al., 2004; Redmond et al., 2006).

Gijon-Nogueron et al. (2016) investigated the FPI distribution score and its variations linked to age in more than 1,500 healthy children, thus setting the reference values for children. The CMT cohort investigated by Redmond et al. (2008) showed a correlation between FPI-6 values and age, with significantly higher FPI-6 scores in the young and the elderly compared to the adult population with a 'U' shaped distribution curve. The availability of normative values of FPI-6 is a further element favoring the adoption of this scale (Redmond et al., 2008).

Most of the studies considered in this review stated the role and the level of expertise of the assessors using FPI-6 (see Table 2). A variety of healthcare professionals were present including physicians, physiotherapists, podiatrists, and physiotherapy and osteopathy students. Significant score differences arose based on the difference in expertise levels. Both intra-rater and inter-rater reliability increased after a short training period. This proves the need for a short training period, requiring about 20-30 supervised evaluations (Cornwall et al., 2008; Evans et al., 2012), further proving the validity of FPI-6. The most difficult items to be properly assessed were those related to the differences between the neutral and pronated foot types (McLaughlin et al., 2016). Following a brief learning period, FPI-6 proved to be a satisfactory tool in all the studies considered: intra-rater reliability results were very good among studies, with intra-rater ICC > 0.90 (Evans et al., 2012; Terada et al., 2014; Kirmizi et al., 2020; Patel et al., 2020) or Cohen's k > 0.85 (Zuil-Escobar et al., 2019) or Pearson's r ≥ 0.89 (Oleksy et al., 2010). The inter-rater ICC varied among the studies, ranging from fair to very good when untrained or trained raters were respectively included (see Table 2) (Menz, 2006; Cornwall et al., 2008; Evans et al., 2012; Griffiths and McEwan, 2012; Terada et al., 2014; Evans and Karimi, 2015; Tucker et al., 2015; McLaughlin et al., 2016; Aquino et al., 2018; Kenny et al., 2018; Hegazy et al., 2020; Kirmizi et al., 2020; Patel et al., 2020). To support operator training, Kirmizi and colleagues suggested implementing the FPI-6 operative manual by including drawings that fully described each possible foot deviation and its associated score (Kirmizi et al., 2020).

A current limitation with FPI-6 is the lack of studies addressing its responsiveness to change after a treatment. Internal responsiveness is the ability to detect a change between the pre- and post-intervention condition, and external responsiveness is the ability to detect a change that truly affects the patient's health status (Menz et al., 2014). The sensitivity of the scale to changes was addressed by Redmond et al. (2006) by applying wedges under specific parts of the foot and verifying the modification in the score. However, no studies specifically designed to assess FPI-6 responsiveness are available. The lack of this information is a current limitation of the scale and should be addressed by future studies.

The natural evolution of CMT is characterized by atrophy of the intrinsic foot muscles and their imbalance with the antagonist extrinsic foot muscles. This leads to foot deformity and a progressive decrease in ankle range of motion (Burns, 2006). Foot deformity can be tracked and quantified by using the FPI-6. Since FPI-6 supplies a measurement for foot alignment alone, this should be combined with other scales addressing further domains related to the patient's functionality.

In light of these observations, we suggest using FPI-6 to assess foot deformities and measuring the patient's functional and impairment levels by using specific tools developed for neuropathies, such as the Charcot-Marie-Tooth Disease Pediatric Scale in Children (Burns et al., 2012b), the CMT Neuropathy Score (Burns et al., 2012b; Zuccarino et al., 2020) along with measures of strength, pain, balance and walking ability. In clinical practice, it can be used to follow the evolution of the cavovarus foot deformity in CMT patients and to assess the effect of foot surgery in restoring the physiological tibiotarsal and foot joint posture.

In research studies, when algebraic operations are required such as the computation of longitudinal differences or the computation of the ensemble average, the use of the linear, numerical version of the FPI-6 score obtained by the Rasch Analysis procedure (Redmond et al., 2006) is advisable.

### Strengths and Limitations

This is the first scoping review about scales used for the clinical evaluation of foot deviations in CMT patients to be found in literature. The main strengths of this review are the comprehensive analysis of the development processes and the psychometric properties of the scales, including a discussion of both their usability and learning curve when available.

The main limitation is that CMT is a rare disease, so studies on this topic compared to other pathologies are few. Moreover, differently from systematic reviews, protocols for scoping reviews cannot be uploaded on dedicated repositories, such as the PROSPERO database (Page et al., 2018). Consequently, a preliminary peer-review of the procedures we used in this scoping review is missing. Even if the string search was built following an iterative process aimed at improving the sensibility of the search, as suggested by scoping reviews guidelines, we could have missed some papers during the database search.

In this study, we did not control for eventual methodological errors in the included studies, according to the procedure for scoping reviews. For managing this limitation, readers are invited to always pay attention to sample numerosity reported in Table 2 and to the use of the proper statistical indicator when assessing the scale metric properties.

## Conclusions

The results of our scoping review suggest the adoption of FPI-6 for foot assessment in the CMT population. The scale demonstrated a high applicability in different cohorts, good psychometric properties, uni-dimensionality, and the ability to differentiate between single patients. FPI-6 requires a short training period for the assessors. We suggest its use in clinical practice as it can be a helpful tool for clinicians in assessing foot deformities in the CMT population, along with functional scales, specifically designed for patients with CMT or similar neuropathies, and suited to assess the patient functional and impairment levels. Future studies should address the responsiveness of FPI-6 when used with different treatments delivered to specific cohorts of patients.

## Data Availability Statement

The original contributions presented in the study are included in the article/supplementary material, further inquiries can be directed to the corresponding author.

## Author Contributions

CR, AM, DM, and SM contributed to conception and design of the study. AM, PP, and FM organized the database. CR and AM wrote the first draft of the manuscript. DM, MG, GB, and PZ wrote sections of the manuscript. All authors contributed to manuscript revision, read, and approved the submitted version.

## Funding

This study was entirely funded by our Institution.

## Conflict of Interest

The authors declare that the research was conducted in the absence of any commercial or financial relationships that could be construed as a potential conflict of interest.

## Publisher's Note

All claims expressed in this article are solely those of the authors and do not necessarily represent those of their affiliated organizations, or those of the publisher, the editors and the reviewers. Any product that may be evaluated in this article, or claim that may be made by its manufacturer, is not guaranteed or endorsed by the publisher.

## Abbreviations

AOFAS-AHES: American Orthopedic Foot & Ankle Society's Hindfoot Evaluation Scale
CMT: Charcot Marie Tooth
FFI: Foot Function Index
FHSQ: Foot Health Status Questionnaire
FPI: Foot Posture Index
FPI-6: Foot Posture Index-6 items
ICC: Intraclass Correlation Coefficient
MFS: Maryland Foot Score
SF-36: Medical Outcomes Study Questionnaire Short Form 36 Health Survey
WSG: Wicart- Seringe grade.
